# Long‐term outcome of high‐grade encapsulated papillary carcinoma of the breast: implications for staging and management

**DOI:** 10.1111/his.70240

**Published:** 2026-08-03

**Authors:** Milena Stevanovic, Emad Rakha, Ian O. Ellis, Umberto Maccio, Bärbel Papassotiropoulos, Hisham Fansa, Lidija Stevanovic, Heike Frauchiger‐Heuer, Isabell Witzel, Zsuzsanna Varga

**Affiliations:** ^1^ Department of Pathology and Molecular Pathology University Hospital Zurich Zurich Switzerland; ^2^ School of Medicine University Nottingham Nottingham UK; ^3^ Department of Histopathology Nottingham University Hospitals NHS Trust Nottingham UK; ^4^ Breast Center Seefeld Zurich Switzerland; ^5^ Breast Center, Hospital Zollikerberg Zollikerberg Switzerland; ^6^ Breast Center, Department of Gynecology University Hospital Zurich Zurich Switzerland; ^7^ Comprehensive Cancer Center University Hospital Zurich Zurich Switzerland; ^8^ University of Zurich Zurich Switzerland

**Keywords:** adverse features, breast, encapsulated papillary carcinoma, high grade, staging, therapeutic management

## Abstract

**Background:**

Encapsulated papillary carcinoma (EPC) of the breast is an uncommon tumour with controversial biological behaviour and staging. Emerging evidence suggests that EPC with adverse pathological features (EPC‐A) may exhibit invasive potential. The absence of uniform staging criteria contributes to management variability. This study evaluates the clinicopathological characteristics and clinical course of high‐grade EPC (HG‐EPC), including EPC‐A.

**Materials and methods:**

This retrospective multicentre study (2000–2025) included 25 HG‐EPC cases, irrespectively of ER/PR and HER2 status. Tumours with frank stromal invasion beyond microinvasive foci were excluded. Histopathological re‐evaluation was performed in accordance with 2019 WHO classification criteria.

**Results:**

Of 38 cases reviewed, 25 fulfilled the inclusion criteria as EPC (mean age 57.2 years, mean tumour size 2.9 cm). Six cases (24%) demonstrated conventional‐type microinvasive components. A subset of 16 cases fulfilled criteria for EPC‐A, defined by high‐grade morphology in combination with ER/PR‐negative (or ER <30% positive) and/or HER2‐positive status. Staging and management were variable. Initial pathological stage varied: pTis (*n* = 13) to pT1 (*n* = 4) or pT2 (*n* = 7), and pT3 (*n* = 1). Adjuvant chemotherapy and radiotherapy were administered in 7 and 12 patients respectively. During follow‐up, four local recurrences (three invasive and one *in situ*) were observed. One of the locally recurrent patients subsequently developed distant metastasis, and one breast cancer‐related death occurred 200 months after diagnosis. Recurrence or death occurred in 20% (5/25) of patients despite therapy. Microinvasion was seen only in the EPC‐A tumours.

**Conclusion:**

HG‐EPC and EPC‐A overlap morphologically and diagnostically with frankly invasive carcinomas and demonstrate a non‐negligible risk of recurrence. Clinical outcomes and more frequent microinvasion in EPC‐A support the view that EPC‐A is more appropriately regarded as an indolent form of invasive carcinoma rather than a purely *in situ* lesion and demonstrate a non‐negligible risk of recurrence and highlight the need for standardized staging criteria.

## Graphical abstract

In the original study by Rakha *et al.*, the authors proposed that high‐grade EPC should be considered within an invasive framework regardless of hormone receptor status. In the present study, we adopted a more selective definition of EPC‐A, restricted to tumours showing more adverse features, including high‐grade morphology together with triple‐negative (or ER positive <30%) or HER2‐positive phenotype, rather than high‐grade morphology alone. Accordingly, high‐grade EPCs that retain an ER‐positive/HER2‐negative phenotype remain of uncertain biological significance and warrant further study. Using these stricter criteria, 25 of 38 cases (66%) met the definition of HG EPC and the inclusion criteria, including 16 cases that showed a combination of HG with TN (or ER positive >30%) or HER2 phenotypes, and these were classified as EPC‐A. HG‐EPC and EPC‐A overlap morphologically and diagnostically with frankly invasive carcinomas and demonstrate a non‐negligible risk of recurrence. Clinical outcomes and the more frequent microinvasion of EPC‐A but not in HG‐EPC support the view that EPC‐A is more appropriately regarded as an indolent form of invasive carcinoma rather than a purely *in situ* lesion and demonstrate a non‐negligible risk of recurrence and highlight the need for standardized staging criteria.

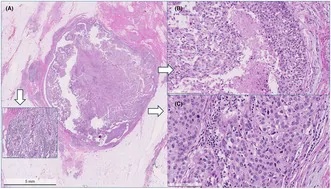

**Abbreviations**
BM: basement membrane
EPC: encapsulated papillary carcinoma
EPC‐A: EPC with adverse pathological features
ER: oestrogen receptor
H&E: haematoxylin and eosin
HG‐EPC: high‐grade EPC
PR: progesterone receptor
SD: standard deviation

## Introduction

Breast cancer remains a leading cause of cancer‐related mortality globally.^1^, ^2^, ^3^ Among its diverse morphological presentations, papillary carcinoma is a rare subtype, accounting for less than 2% of all breast malignancies.^1^, ^2^, ^4^, ^5^, ^6^, ^7^ Characterized by malignant epithelial proliferation supported by fibrovascular cores, these tumours present significant diagnostic and classification challenges due to their overlapping morphological features and varied biological potential.^2^, ^8^, ^9^, ^10^, ^11^, ^12^, ^13^

Papillary carcinomas of the breast can be categorized into several subtypes, including papillary ductal carcinoma *in situ* (papillary DCIS), solid papillary carcinoma, encapsulated papillary carcinoma (EPC) and invasive papillary carcinoma.^1^, ^2^ EPC, previously referred to as intracystic or encysted papillary carcinoma, is characterized by delicate fibrovascular cores lined by neoplastic epithelial cells of low to intermediate nuclear grade, surrounded by a well‐developed fibrous capsule and typically lacking myoepithelial cells within the cores and at the periphery.^1^, ^2^ EPC has traditionally been regarded as an *in situ* carcinoma, largely due to its indolent clinical behaviour^11^, ^14^ and the hypothesis that the surrounding fibrous capsule represents a modified or proliferative basement membrane (BM), supported by collagen IV expression.^6^, ^8^, ^11^, ^13^ However, this concept was challenged by some authors.^15^, ^16^, ^17^ The absence of myoepithelial cells at the epithelial–stromal interface of a malignant breast lesion generally supports an invasive nature.^18^ Moreover, the significance of the surrounding fibrous capsule in EPC remains controversial. Some authors have proposed that it represents a reactive stromal response formed around the pushing, circumscribed borders of a slowly growing malignant cystic neoplasm, and therefore does not reliably exclude invasion.^19^, ^20^ Notably, similar fibrous encapsulation has also been described in recurrent EPC and in metastatic deposits, further supporting the concept that the capsule is not a definitive marker of *in situ* disease.^2^, ^11^, ^12^, ^14^

Despite these emerging biological considerations, the 2019 WHO working group^2^ elected to retain the *in situ* staging framework to avoid overtreatment, even in the presence of features suggestive of limited invasive potential or occasional reports of metastatic spread.^2^, ^11^, ^12^, ^14^ Overall, the cumulative evidence including well‐circumscribed morphology, a luminal phenotype, low nuclear grade, the presence of a BM‐like capsule and the rarity of adverse outcomes supports maintaining *in situ* staging for EPC, while acknowledging its unique and intermediate biological profile.^2^, ^11^, ^12^, ^14^

However, with the accumulation of additional cases, it has become evident that a subset of these tumours exhibits adverse prognostic features (EPC‐A), including high nuclear grade, a phenotype that is typically triple negative (oestrogen receptor (ER), progesterone receptor (PR) and HER2 negativity) or ER positive (<30%), and/or HER2 positive, and an increased frequency of focal capsule irregularities or invasion‐like patterns.^4^, ^21^ These EPC‐A cases appear to harbour cumulative features suggestive of invasive potential, making it increasingly difficult to justify a uniform *in situ* staging approach.^4^, ^12^, ^21^ Furthermore, distinguishing such high‐risk tumours that lack myoepithelial cells from other circumscribed (EPC‐like) forms of invasive breast carcinoma requires clear biological and clinical justification, as well as reproducible criteria. Circumscription and/or papillary architecture alone are insufficient to justify downstaging from invasive to *in situ* disease, despite their association with a favourable prognosis.^21^, ^22^, ^23^, ^24^, ^25^

In 2015, Rakha, Varga *et al*. suggested that high‐grade EPC may represent a non‐conventional invasive form of breast cancer and should therefore be managed similarly to invasive breast carcinoma.^12^ Nevertheless, long‐term follow‐up studies confirming this hypothesis remain limited, and the concept continues to be debated.^13^, ^15^, ^17^, ^24^, ^26^ In addition, it remains unclear whether such tumours should be classified as high‐grade EPC or more specifically as EPC with adverse features (EPC‐A), defined by a combination of high‐grade morphology and the triple‐negative or ER‐positive (<30%) and/or HER2‐positive phenotype. This constellation of adverse features may more robustly identify a subset with clinically meaningful risk, supporting consideration of classification and staging as invasive disease.

The present study evaluates the long‐term outcomes in patients with high‐grade EPC, including EPC‐A. The primary endpoint was a descriptive analysis of long‐term outcomes in patients with EPC‐A, including local (*in situ* or invasive) and distant recurrence, development of second primary carcinomas, and mortality. The secondary endpoint was to assess whether the management of high grade EPC including EPC‐A in this cohort corresponded to the recommendations proposed by Rakha *et al*. and the 2019 WHO classification of breast tumour (i.e. the whole tumour considered invasive), earlier classifications (i.e. the whole tumour considered *in situ*) or reflect individual interpretative judgement by the reporting pathologist (i.e. only part of the tumour considered invasive to avoid overtreatment).^2^, ^12^

## Materials and methods

### Patient Cohort

A retrospective search of pathology reports from the Department of Pathology and Molecular Pathology at the University Hospital Zurich, Switzerland, was performed for the period between 2000 and 2023. Inclusion criteria were:

- histological diagnosis of encapsulated papillary carcinoma on review
- limitation of invasion to microinvasive foci (max. 1 mm)
- high nuclear grade (threefold variation in size and coarse chromatin) at least in a subset of the lesion
- female sex.

Oestrogen and progesterone receptor status (in all patients) and HER2 status (via IHC and/or ISH) when available were included if the above‐mentioned morphological criteria were fulfilled.

EPC‐A (EPC with adverse prognostic features) was defined as ECP with high nuclear grade and triple‐negative (ER/PR and HER2 negativity) or ER‐positive (<30%), and/or HER2‐positive phenotype.

HG‐EPC was defined as ECP with high nuclear grade and luminal phenotype (ER/PR >30% and HER2 negative if HER2 status was available).

The TNM staging of the primary tumours had originally been determined and also discussed during interdisciplinary tumour board meetings. For this study, the TNM stages documented in the tumour board protocols were used.

In addition to newly identified cases, previously reported cases by Rakha, Varga *et al*. were re‐evaluated.^12^ Furthermore, additional cases meeting the above‐mentioned criteria were identified from the consultation service of the Department of Pathology, Breast Center, University Nottingham and Nottingham University Hospitals NHS, United Kingdom.

### Patient Characteristics

Thirty‐eight patients were identified; however, one patient had to be excluded due to lack of complete clinicopathological staging, and 12 patients were excluded after a retrospective re‐evaluation of the conventional histological diagnosis in each case. The excluded cases all exhibited overt invasive growth beyond microinvasive foci, so these cases were not classified as ECP‐A. They were considered as invasive breast carcinoma with EPC‐like pattern as the basic pattern of a cystic papillary lesion was visible. This resulted in a cohort of 25 patients that fulfilled the inclusion criteria. Haematoxylin and eosin (H&E)‐stained specimens were available for all these 25 cases, and immunohistochemical slides for ER/PR and HER2 were available for most of them. Clinical and follow‐up data were retrieved using the clinical information system of the University Hospital Zurich Switzerland and its affiliated breast centres (Breast Center Seefeld and Breast Center of the Hospital Zollikerberg) and from the available clinicopathological charts from the Department of Pathology, Breast Center, University Nottingham and Nottingham University Hospitals NHS, United Kingdom. Data on patient age, age at diagnosis, tumour size, histological subtype, lympho‐vascular invasion and ER‐, PR‐ and HER2‐status were collected. In eight cases, missing ER, PR and/or HER2 status were reassessed retrospectively from available tumour tissue in FFPE (formalin‐fixed, paraffin‐embedded) tissue in most cases. Clinical treatments were categorized as either surgery (mastectomy vs. breast‐conserving surgery, resp. segmentectomy) or adjuvant therapy (chemotherapy and radiotherapy). Follow‐up time was dated from the last documented contact with the patient in the clinical information system.

### Statistics

All statistical analyses were performed using R software version 4.1.2 (The R foundation for Statistical Computing, Vienna, Austria). Results are expressed as mean ± standard deviation (SD) and statistical significance was considered at *P*‐value <0.05 with a confidence interval of 95%. The applied codes are available to readers upon request.

### Ethics

This study was a part of a larger breast cancer study previously approved by the Ethical Committee of Canton Zurich (KEK‐2012‐553, PB‐2019‐00111, BASEC 2024‐01804). The use of clinical and pathological data from the cohort, including the consent procedures governing their use in this study, was approved by the Ethics Committee of the Canton of Zurich.

## Results

### Patient Characteristics

Twenty‐five cases met the previously described characteristics (13 cases were excluded as described above). Patients' ages at diagnosis ranged from 23 to 82 years, with an average of 57.17 ± 17.13 years. The size of the primary tumour ranged from 0.5 cm to 8 cm, with an average of 2.89 ± 2.09 cm. The exact tumour size could not be retrieved for one patient (No. 16), but it was staged as pTis. The original pathology reports staged 12 cases as *in situ* carcinoma (pTis), two as pT1a, two as pT1c and seven as pT2. One case was staged as pT3 as the whole tumour was considered as invasive. Ten patients had triple‐negative or ER‐positive (<30%) tumours. HER2 was positive (either scored 3+ or amplified in FISH) in six cases. HER2 status was incompletely available or unavailable in six patients. Nodal status was available in 21 patients (either sentinel node or axillary dissection depending on the original staging); one patient (No. 19) had isolated tumour cells in the sentinel lymph nodes (ITC, i+).

All characteristics of the cohort are summarized in Table 1.

**Table 1:** Clinicopathological characteristics of the cohort

Patient's number	Age at first diagnosis (years)	Tumour size (cm)	Grading	pT (as staged in original report)	Revised histological diagnosis	ECP biological subgroup	pN	R‐status	ER/PR‐status	HER‐2 status (IHC/FISH)	Ki67 index	Surgery segmentectomy	Surgery mastectomy	Chemotherapy	Endocrine therapy	Radiotherapy
1^a^	52	4.2	High grade	pTis	EPC	EPC‐A	pN0	R0	Negative/negative	Score 0	60%	Yes	No	Yes	No	Yes
2^a^	44	6.5	High grade	pT3	EPCmi	EPC‐A	pN0	R0	Negative/negative	Score 1+	70%	Yes	No	Yes	No	Yes
3	44	4.5	High grade	pT2	EPCmi	EPC‐A	pN0	R0	Negative/negative	Score 0	40%	Yes	No	Yes	No	Yes
4	39	3	High grade	pT2	EPC	EPC‐A	pN0	R0	Negative/negative	Score 0	70%	Yes	No	Yes	No	Yes
5	66	2.5	High grade	pTis	EPC	EPC‐A	pNx	R0	Negative/negative	Score 0	25%	Yes	No	No	No	Yes
6	40	3.5	High grade	pTis	EPC	EPC‐A	pN0	R0	Negative/negative	Score 0	NA	Yes	No	Yes	No	Yes
7	74	0.5	High grade	pTis	EPC	EPC‐A	pNx	R0	30%/negative	*NA*	NA	Yes	No	No	No	No
8	68	1.3	High grade	pT1a	EPCmi	EPC‐A	pN0	R0	20%/negative	Score 2+/ FISH neg	40%	Yes	No	No	No	Yes
9	38	1.2	High grade	pT1c	EPCmi	EPC‐A	pN0	R0	Negative/negative	Score 1+	25%	Yes	No	*No*	No	Yes
10	58	1.5	High grade	pT1c	EPC	EPC‐A	pN0	R0	Negative/Negative	Negative	NA	No	Yes	NA	No	NA
11^a^	68	3.2	High grade	pT2	EPCmi	EPC‐A	pN0	R0	Negative/negative	Score 3+	40%	Yes	No	Yes	No	Yes
12	80	7	High grade	pTis	EPC + Paget	EPC‐A	pN0	R0	Negative/negative	Score 3+	NA	No	Yes	No	No	No
13	54	1.3	High grade	pTis	EPC	EPC‐A	pN0	R0	100%/30%	Score 3+	NA	Yes	No	No	No	Yes
14	39	2.5	High grade	pT2	EPCmi	EPC‐A	pN0	R0	20%/2%	Score 2+/ FISH +	80%	No	Yes	Yes	Yes	No
15	54	0.5	Intermediate + high grade	pT1a	EPC	EPC‐A	pNx	R0	Negative/negative	Score 3+	NA	Yes	No	Yes	No	No
16	79	*NA*	Intermediate + high grade	pTis	EPC	EPC‐A	pNx	R0	100%/negative	Score 3+	NA	No	Yes	*NA*	*NA*	*NA*
17	79	0.8	High grade	pT2	EPC	HG‐EPC	pN0	R0	100%/negative	Score 0	NA	No	Yes	No	No	No
18	39	1.5	High grade	pTis	EPC	HG‐EPC	pNx	R0	100%/100%	Negative	NA	Yes	No	No	Yes	Yes
19^a^	59	8	High grade	pTis	EPC	HG‐EPC	pN0 (i+)	R0	100%/100%	Score 1+	NA	No	Yes	No	Yes	No
20	70	1.6	High grade	pTis	EPC	HG‐EPC	pN0	R0	100%/70%	Score 2+/FISH NA	NA	No	Yes	No	No	No
21	49	2.5	High grade	pTis	EPC	HG‐EPC	pN0	R0	80%/20%	Score 2+/FISH NA	NA	Yes	No	No	No	Yes
22	23	1.3	High grade	pTis	EPC	HG‐EPC	pN0	R0	100%/5%	Score 2+/FISH NA	NA	No	Yes	No	No	No
23	75	3.5	Intermediate + high grade	pT2	EPC	HG‐EPC	pN0	R0	100%/negative	*NA*	NA	No	Yes	*NA*	*NA*	*NA*
24	82	2.5	Intermediate + high grade	pTis	EPC	HG‐EPC	pNx	R0	100%/<10%	*NA*	NA	Yes	No	*NA*	*NA*	*NA*
25	68	2.2	HIGH grade	pT2	EPC	HG‐EPC	pN0	R0	80%/NA	NA	70%	No	Yes	No	NA	No

ECP + Paget, encapsulated papillary carcinoma with M. Paget; ECP‐A, encapsulated papillary carcinoma with adverse pathological features; ECPmi, encapsulated papillary carcinoma with microinvasion; EPC, encapsulated papillary carcinoma; HG‐EPC, high‐grade encapsulated carcinoma, luminal type; NA, not available.

^a^ Case published in *Histopathology*, Rakha *et al*. 2015.^12^

### Surgical Modalities/Therapies

Of the 25 patients identified, 10 underwent mastectomy and 15 had breast conserving surgery (wide local excision or segmentectomy). Of the 25 patients, 8 received chemotherapy, including 1 patient (No. 15) who received neoadjuvant chemotherapy due to the contralateral NST carcinoma. Of the 25 patients, 12 received radiotherapy. There was no further information on adjuvant therapy for four patients.

Of the 25 patients, 6 had HER2‐positive disease; 3 of them received anti‐HER2 therapy.

Of the 25 patients, 13 had hormone receptor‐positive disease (3 of them received anti‐hormone therapy).

Therapeutic decisions were made based on the original tumour stage in the primary pathology reports, as well as discussions at the interdisciplinary meeting regarding the biological value of the entity. These discussions influenced the decision to favour adjuvant chemotherapy in cases 1 and 6. For patients 10, 16, 23 and 24, there was no documented information or follow‐up regarding the decision on adjuvant chemotherapy or radiotherapy. It can be assumed that adjuvant therapies were omitted due to the advanced age of patients 16, 23 and 24 (over 75 years old).

The type of surgery and further adjuvant therapies are summarized in Table 1.

### Histological Review of Morphology

All cases underwent histological review by a board‐certified pathologist with expertise in breast pathology (Z.V.), based on the original slides, digital images or newly prepared slides when the original material was unavailable. According to the 2012 and 2019 WHO classification criteria, all 25 cases were classified as high‐grade EPC, characterized by circumscribed borders, papillary architecture and the absence of immunohistochemical evidence of myoepithelial cells, primarily assessed using p63 immunohistochemistry.

All tumours exhibited high‐grade histological features. Cases that, upon review, were considered unequivocally high‐grade invasive carcinomas with features mimicking EPC including circumscription, microvascular arborization and peripheral fibrosis were excluded from the study.

The cohort was heterogeneous with respect to ER, PR and HER2 status. Six cases demonstrated microinvasive foci of conventional no special type (NST) carcinoma, identified on haematoxylin and eosin (H&E)‐stained sections; all microinvasive cases belonged to the EPC‐A category (Figures 1, 2, 3).

**Figure 1:**
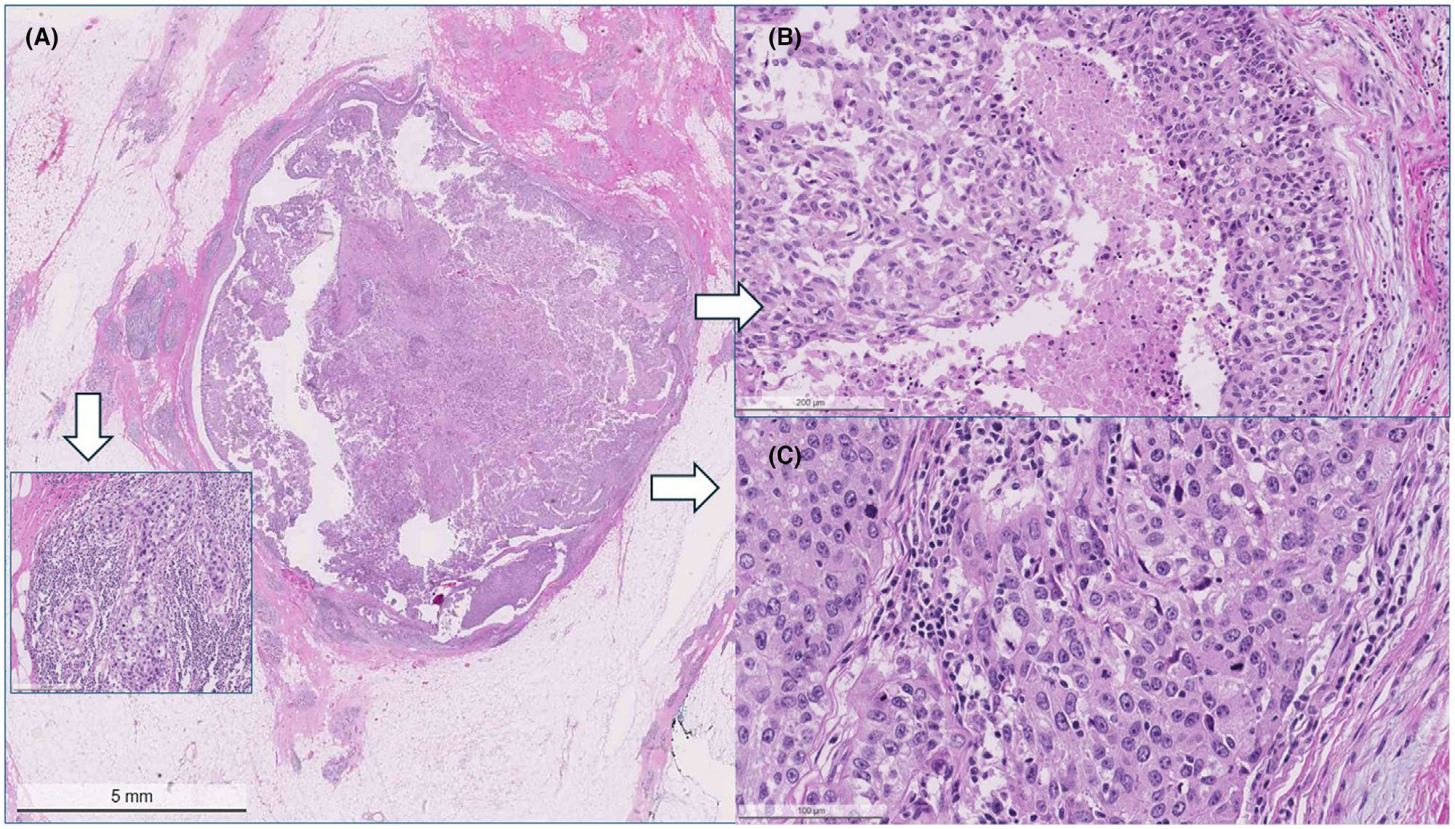
Encapsulated high‐grade papillary carcinoma without invasion. (**A**) Low‐power view: circumscribed papillary growth within a fibrous capsule. Inset shows adjacent conventional DCIS. (**B**) High‐power view of nuclear morphology and papillary architecture with comedo‐type necrosis and high‐grade nuclei. (**C**) High‐grade nuclei with mitotic figures. All images haematoxylin and eosin stain (H&E).

**Figure 2:**
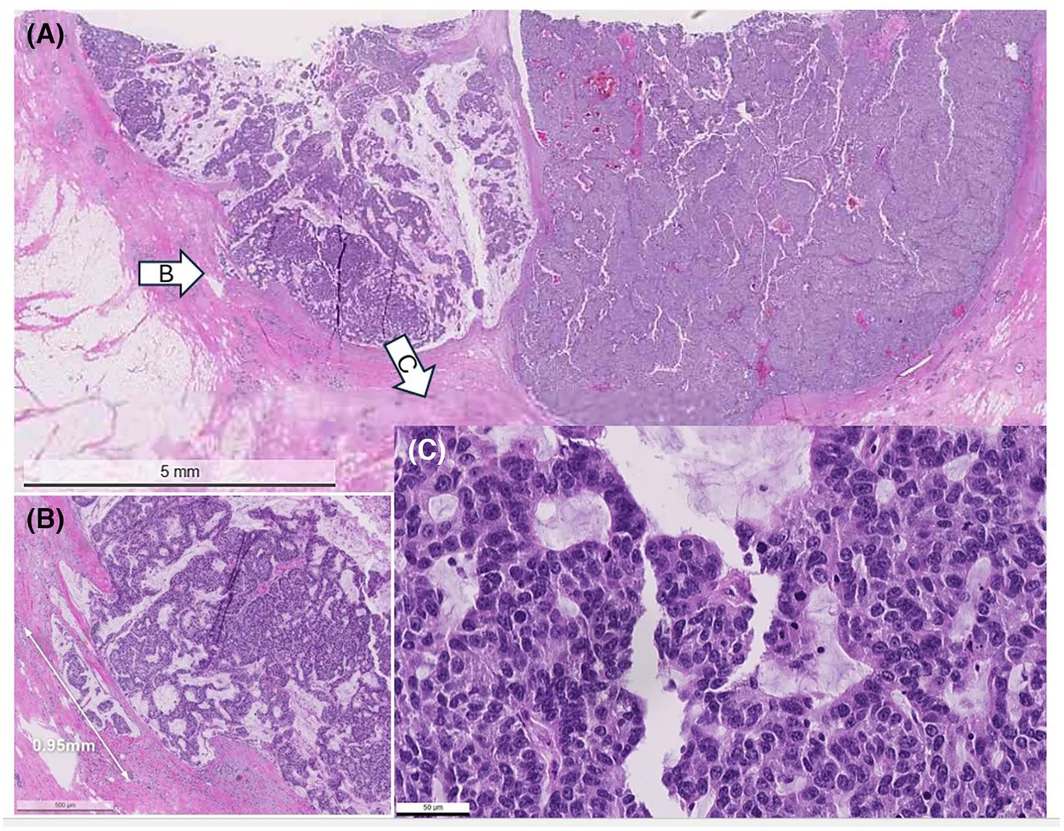
Encapsulated high‐grade papillary carcinoma with microinvasion. (**A**) Low‐power view: circumscribed papillary growth partially with mucin production within a fibrous capsule. (**B**) High magnification of arrow B area with a microinvasive focus (0.95 mm). (**C**) High‐grade nuclei with mitotic figures in the area of arrow (**C**). All images haematoxylin and eosin stain (H&E).

**Figure 3:**
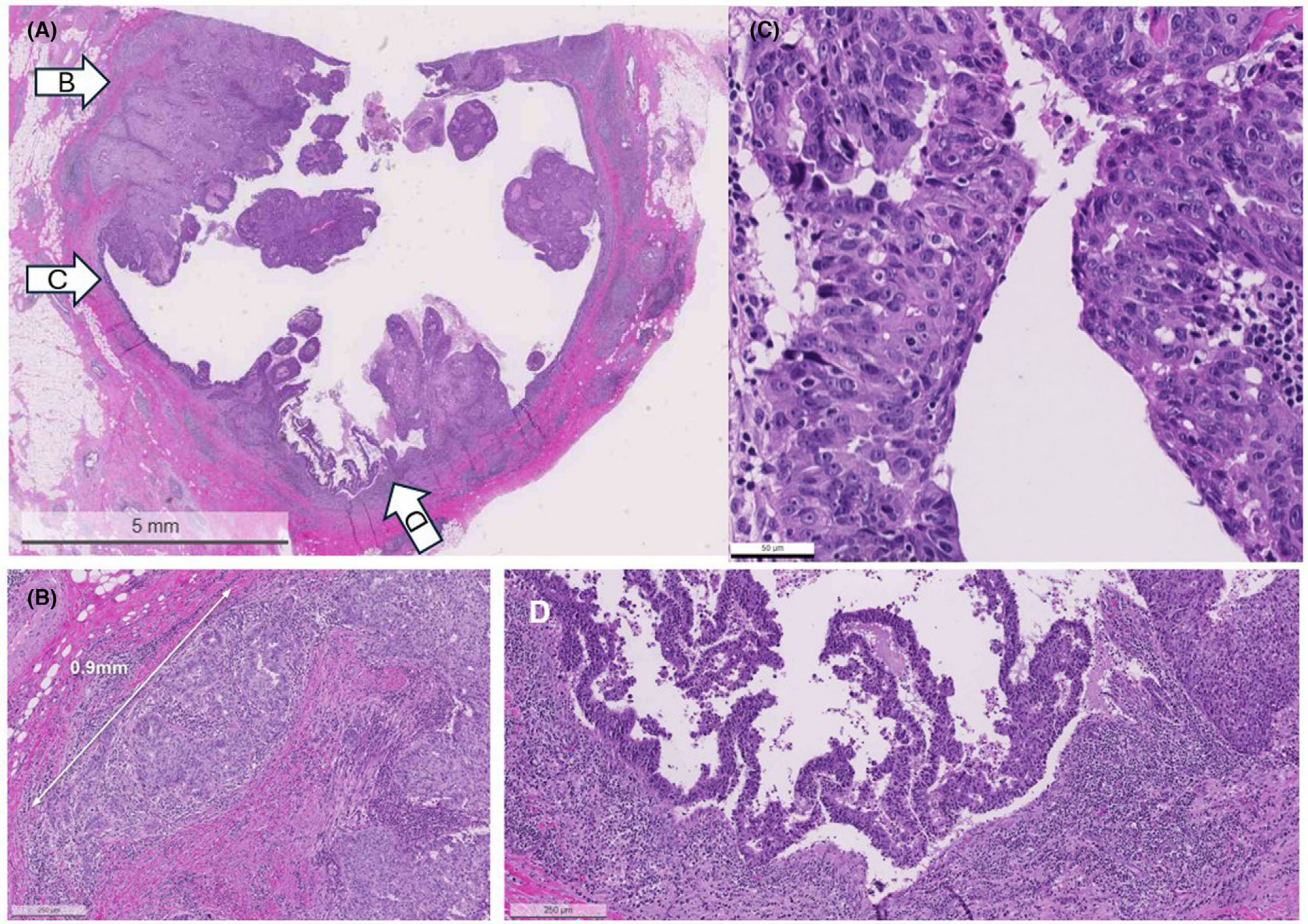
Encapsulated high‐grade papillary carcinoma with a microinvasive focus. (**A**), Low‐power view: circumscribed papillary growth within a fibrous capsule. (**B**) Adjacent to the encapsulated papillary carcinoma an unifocal microinvasive focus (0.9 mm). (**C**) High‐power view circumscribed papillary architecture (**D**) High‐grade nuclei with mitotic figures and a still recognizable papillary architecture. All images haematoxylin and eosin stain (H&E). [Colour figure can be viewed at wileyonlinelibrary.com]

### Outcome

Follow‐up time between 3 and 237 months (median 106 months). A total of five events occurred during the cohort's follow‐up. In these cases, the tumour‐free follow‐up time ranged from 15 to 87 months. Three patients (No. 8, 11 and 22) developed invasive recurrences. One patient (No. 15) developed an *in situ* recurrence during follow‐up. One patient (No. 17) died of breast cancer 200 months after the initial diagnosis.

Patient No. 11 developed contralateral invasive apocrine carcinoma 87 months after the initial EPC diagnosis.

Patient No. 22 developed an ipsilateral invasive NST carcinoma 36 months after an ipsilateral EPC diagnosis.

Patient No. 15 developed an ipsilateral EPC 15 months after being diagnosed with a high‐grade DCIS with pT1a invasive NST components in the same breast. Therefore, the EPC is considered a recurrent *in situ* lesion. This patient was lost to follow‐up after 3 months; however, she was treated with neoadjuvant anti‐Her2 therapy and chemotherapy for the ipsilateral pT1a NST carcinoma, which was also Her2 positive, as was the recurrent EPC.

Patient No. 23 was diagnosed with colorectal carcinoma approximately 15 years after the EPC diagnosis.

Patient No. 8 developed metastatic NST carcinoma 75 months after the EPC diagnosis.

There was no significant correlation between the revised pT stage (pTis vs pTmi), ER/PR/HER2 status and the frequency of events during follow‐up (Table 2; Figure 4).

**Figure 4:**
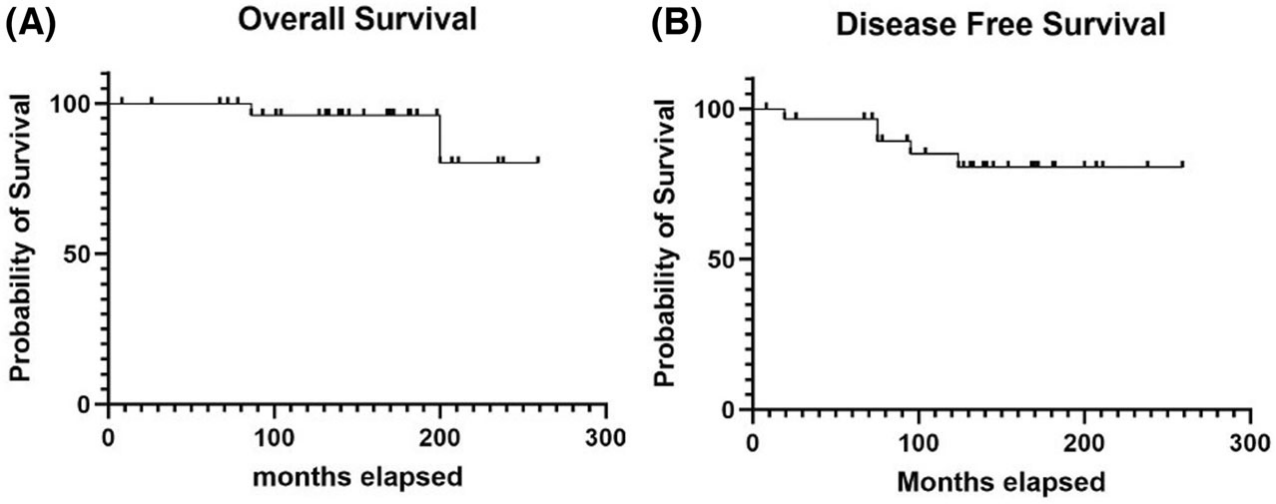
Survival analysis of encapsulated papillary carcinomas. Overall survival (**A**) and disease‐free survival (**B**) estimated the events occurred in the cohort. As there is now a control‐arm in this study, both Kaplan–Meier estimates are solely descriptive without *P*‐values.

**Table 2:** Long‐term survival data characteristics of the cohort

Patient number	Biological EPC subgroup	Revised histological diagnosis	Recurrence *in situ* carcinoma (breast)	Recurrence invasive carcinoma (breast)	Secondary carcinoma, other (non‐mammary)	DFS (follow‐up without tumour manifestation) (months)	Survival until death (months)	ECP treated as invasive carcinoma	Events in follow‐up	Time to follow‐up (months)
1^a^	EPC‐A	EPC	‐	‐	‐	134	‐	Yes	No	134
2^a^	EPC‐A	EPCmi	‐	‐	‐	46	‐	Yes	No	46
3	EPC‐A	EPCmi	‐	‐	‐	116	‐	Yes	No	116
4	EPC‐A	EPC	‐	‐	‐	76	‐	Yes	No	76
5	EPC‐A	EPC	‐	‐	‐	96	‐	No	No	96
6	EPC‐A	EPC	‐	‐	‐	120	‐	Yes	No	120
7	EPC‐A	EPC	‐	‐	‐	169	‐	No	No	169
8	EPC‐A	EPCmi	‐	Yes	‐	75	‐	Yes	Yes	113
9	EPC‐A	EPCmi	‐	‐	‐	38	‐	Yes	No	38
10	EPC‐A	EPC	‐	‐	‐	NA	‐	NA	NA	NA
11^a^	EPC‐A	EPCmi	‐	Yes	‐	87	‐	Yes	Yes	169
12	EPC‐A	EPC + Paget	‐	‐	‐	125	‐	No	No	125
13	EPC‐A	EPC	‐	‐	‐	150	‐	No	No	150
14	EPC‐A	EPCmi	‐	‐	‐	37	‐	Yes	No	37
15	EPC‐A	EPC	Yes	‐	‐	15	‐	Yes	Yes	3
16	EPC‐A	EPC	‐	‐	‐	64	‐	No	No	64
17	HG‐EPC	EPC	‐	‐	‐	‐	200	Yes	No	198
18	HG‐EPC	EPC	‐	‐	‐	237	‐	No	No	237
19^a^	HG‐EPC	EPC	‐	‐	‐	139	‐	No	No	139
20	HG‐EPC	EPC	‐	‐	‐	109	.	No	No	109
21	HG‐EPC	EPC	‐	‐	‐	95	‐	Yes	No	95
22	HG‐EPC	EPC	‐	Yes	‐	36	‐	No	Yes	97
23	HG‐EPC	EPC	‐	‐	Yes (colon cancer)	180	‐	No	Yes	180
24	HG‐EPC	EPC	‐	‐	‐	9	‐	No	No	9
25	HG‐EPC	EPC	‐	‐	‐	24	‐	Yes	No	24

ECP + Paget, encapsulated papillary carcinoma with M. Paget; ECP‐A, encapsulated papillary carcinoma with adverse pathological features; ECPmi, encapsulated papillary carcinoma with microinvasion; EPC, encapsulated papillary carcinoma; HG‐EPC, high‐grade encapsulated carcinoma, luminal type; NA, not available.

^a^ Case published in *Histopathology*, Rakha *et al*. 2015.^12^

## Discussion

In this retrospective study, we analysed a cohort of high‐grade EPC with the aim of better characterizing its biological behaviour. Among the total of 38 cases of high‐grade EPC retrieved and reviewed, 12 cases (32%) showed frank stromal invasive disease sufficient for their reclassification as invasive breast carcinoma with EPC‐like pattern and these were excluded. Among the 25 included cases, 3 cases were >5 cm, 11 cases were 2–5 cm in size and 6 cases showed microinvasive foci of conventional NST carcinomas, as diagnosed by H&E morphology. Twenty per cent of patients developed recurrent disease, including one patient with distant metastasis. Considerable heterogeneity in the originally assigned TNM stages of these tumours was observed, which translated into substantial variation in therapeutic management. This variability highlights the ongoing lack of uniform and universally accepted staging criteria for high‐grade EPC.^2^, ^11^, ^14^, ^27^ The fact that two patients received systemic adjuvant chemotherapy despite their original stage being pTis also reflects variation in management.^2^, ^28^

To contextualize our findings, we included four cases previously reported in the study by Rakha, Varga *et al*., which significantly influenced the perception and clinical management of high‐grade EPC.^12^ That study contributed to the view, later reflected in the 2019 WHO Classification of Breast Tumours, that high‐grade EPC represents a biologically aggressive variant of EPC and may warrant management similar to invasive breast carcinoma.^2^, ^12^ Although classical‐type EPC has historically been regarded as an *in situ* lesion to reflect its indolent clinical behaviour, increasing evidence suggests that certain forms, particularly those with high‐grade cytomorphology showing immunophenotype characteristics of aggressive behaviour, have adverse prognostic factors that argue against applying the same approach in staging.^2^, ^28^ The adverse features together with the frequent association with microinvasion as is the case in our cohort, the identification of increasing cases with metastatic disease (nodal and distant) argue against the stage as *in situ* carcinoma. These tumours are biologically invasive and show features of aggressive biological behaviour, but the available evidence suggests an indolent clinical behaviour.^4^, ^25^, ^27^, ^29^ This concept should be considered in the management algorithms of such tumours rather than on the stage of the tumours that reflects the biological nature of the lesion. The subjectivity of the distinction between such tumours and the aggressive forms of triple‐negative and HER2‐positive invasive breast carcinomas that show circumscribed borders or focal papillary‐like architecture makes the risk of under‐staging of these estimations problematic. Nevertheless, high‐grade EPC‐A in our cohort exhibited a rather heterogenous biological profile in terms of ER/PR/HER2 expression, 16 of them being either triple‐negative, ER‐positive (<30%) or HER2‐positive and 9 cases being luminal‐type tumours.

In routine practice, the distinction of EPC‐A and the classical EPC is easier than the distinction between EPC‐A and EPC‐like invasive carcinoma (personal experience) and also demonstrated by the significant variation in literature regarding the classification of these lesions.^4^, ^25^, ^27^, ^29^

The 5th edition of the WHO classification describes low‐ and intermediate‐grade EPC as a ‘self‐contained indolent invasive carcinoma with a prognosis similar to carcinoma in situ’, recommending staging as pTis (DCIS).^2^ In contrast, cases demonstrating frank invasion, defined by neoplastic cells extending beyond the fibrous capsule with an irregular infiltrative pattern, should be graded using the Nottingham system and staged according to the invasive component.^2^ Furthermore, lesions displaying high‐grade cytological features, increased mitotic activity or unfavourable molecular profiles such as triple‐negative or HER2‐positive status are recommended to be staged and managed as invasive breast carcinoma.^2^, ^4^, ^21^, ^25^, ^30^

The forthcoming 6th edition of the WHO Classification of Breast Tumours readdresses this issue and updates the recommendations for staging in EPC‐A.^31^ The revised guidance states that, for accurate staging and appropriate treatment planning, the possibility that these lesions represent pseudopapillary high‐grade invasive carcinomas mimicking EPC should be carefully assessed and either confirmed or excluded. Multidisciplinary review is recommended to facilitate accurate diagnosis, particularly tumour staging and to guide optimal patient management.^31^

However, as this recommendation is based on a limited number of cases, debate about the staging of such tumours continued. The aim of this study has been to increase the evidence base to support or refute such an important recommendation. Concerns that some cases of high‐grade invasive carcinoma which mimic high‐grade EPC could be misclassified as EPC and not receive appropriate treatment were raised. Such cases were excluded from this study on rigorous pathology review.

In the original study by Rakha *et al*., the authors proposed that high‐grade EPC should be considered within an invasive framework regardless of the receptor status.^12^ In the present study, we on purpose adopted a more selective definition of EPC‐A, restricted to tumours showing more adverse features, including high‐grade morphology preferably together with triple‐negative or HER2‐positive phenotype, rather than high‐grade morphology alone. However, on review, we identified a subset of luminal‐like EPC‐like tumours exhibiting high‐grade papillary morphology, which we also included into this cohort. Accordingly, high‐grade EPCs that retain an ER‐positive/HER2‐negative phenotype remain of uncertain biological significance and warrant further comparative and clinicopathological studies. Using these stricter criteria, 25 of 38 cases (66%) met the definition of HG EPC and the inclusion criteria, including 14 cases that showed a combination of HG with TN or HER2 phenotypes, and these were classified as EPC‐A and 10 cases which retained ER positivity.

The tumours included in our cohort displayed exclusively features of the high‐grade disease form of ECP. Notably, the original TNM classifications assigned to some of these cases were performed prior to the introduction of the 2019 WHO criteria or were interpreted differently.^2^ Re‐evaluation of the specimens according to 2019 WHO guidelines revealed discrepancies between the original staging and the staging that would be assigned today. These differences likely reflect both the historical lack of standardized criteria and the inherent subjectivity in interpreting the invasive potential of EPC.^2^, ^12^

This staging ambiguity has important clinical consequences. Whether a lesion is classified as *in situ or invasive directly determines the indication for adjuvant therapies*, including radiotherapy, endocrine therapy, chemotherapy and axillary staging procedures.^28^, ^32^ Lesions staged as DCIS are typically managed with local therapy and, when indicated, endocrine treatment, whereas invasive carcinomas may require systemic therapy and nodal staging.^28^, ^32^ As a result, patients with morphologically similar tumours may receive markedly different treatment regimens depending on the staging interpretation.

This variability in staging and treatment also complicates the interpretation of long‐term outcomes. Because patients are managed according to different therapeutic strategies, it becomes difficult to determine whether recurrence rates or disease‐free outcomes reflect the intrinsic biology of high‐grade EPC or differences in treatment intensity. Consequently, stratification of follow‐up data according to stage is inherently challenging, particularly in small cohorts such as ours. Notably, similar fibrous encapsulation has also been described in recurrent EPC and in metastatic deposits, further supporting the concept that the capsule is not a definitive marker of *in situ* disease.^11^, ^14^, ^18^, ^27^

Despite these limitations, the overall mortality in our cohort was low, with only one recorded death. The observed recurrence rate of 16% is comparable to that reported for DCIS, further emphasizing the unresolved question of whether high‐grade EPC behaves more like an *in situ* lesion or a low‐grade invasive carcinoma.^2^, ^15^, ^26^, ^28^, ^32^, ^33^, ^34^, ^35^

We were able to extend the follow‐up of several cases originally reported in 2015 and complement them with additional cases.^12^ Nevertheless, the follow‐up intervals varied substantially among patients, and the rarity of EPC limited the size of the dataset. To enable meaningful analysis, we therefore included EPC cases also with microinvasion after review. A strength of the study is that all pathological specimens were evaluated or re‐evaluated at the same institution, the Department of Pathology and Molecular Pathology at the University Hospital Zurich, allowing consistent re‐evaluation of histological features alongside access to the corresponding clinical data.

## Conclusion

Our study highlights the ongoing controversy surrounding the staging and classification of EPC‐A. In routine practice, these tumours overlap morphologically and diagnostically with frankly invasive carcinomas, and a subset may be classified as invasive breast carcinoma on review. The findings support a risk‐adapted approach, recognizing that EPC‐A should not be uniformly regarded as *in situ* carcinoma and may be more appropriately considered an indolent form of invasive carcinoma while taking its relatively favourable prognosis into account when planning management. Although this study does not fully resolve the debate, it emphasizes the need for clearer, more reproducible diagnostic and staging criteria. Such standardization is essential to ensure consistent clinical management and to enable meaningful comparison of outcomes in future studies. Given the rarity and complexity of these lesions, the authors strongly emphasize the need for large international multicentre studies to validate current staging criteria and management recommendations. Such collaborative efforts are crucial for improving diagnostic reproducibility, advancing understanding of tumour biology and clinical behaviour, and establishing robust evidence‐based guidelines for patient management.

## Author contributions

M.S. collected the data, drafted the manuscript and visualized the results. E.R. conceived and designed the study, drafted the manuscript, contributed to the data collection and critically revised the manuscript. I.O.E. critically revised the manuscript. U.M. critically revised the manuscript. B.P. contributed to the data collection and critically revised the manuscript. H.F. critically revised the manuscript. L.S. critically revised the manuscript and visualized results. H.F.‐H. critically revised the manuscript. I.W. critically revised the manuscript. Z.V. conceived and designed the study, supervised the project, drafted the manuscript and critically revised the manuscript. All authors read and approved the final manuscript.

## Funding information

No funding was necessary for this study.

## Conflict of interest statement

There is no conflict of interest to declare for each author.

## Data Availability

The data that support the findings of this study are available on request from the corresponding author. The data are not publicly available due to privacy or ethical restrictions.
